# Malignant seeding of the biopsy needle tract outside of the radiation therapy field in a patient with Glioblastoma

**DOI:** 10.1259/bjrcr.20190070

**Published:** 2020-09-29

**Authors:** Maxwell E. Cooper, Jim Zhong, Sera Kim, Michael J. Hoch, Brent D. Weinberg

**Affiliations:** 1 Department of Radiology and Imaging Sciences, Emory University School of Medicine, Atlanta, GA; 2 Department of Radiation Oncology, Emory University School of Medicine, Atlanta, GA; 3 Department of Radiology, University of Pennsylvania School of Medicine, Philadelphia, PA

## Abstract

A 44-year-old male initially presented with a right thalamic brain tumor that was confirmed with stereotactic biopsy to be glioblastoma (GBM). The patient was treated with radiotherapy and temozolomide for 6 weeks. At 1 month after completing chemoradiation therapy, the patient underwent follow-up imaging that revealed the primary lesion had mildly responded to chemoradiation, but a secondary lesion had developed along the biopsy needle tract. This secondary lesion was outside of the field of radiation therapy for the primary tumor and concluded to be intracranial spread of GBM along the biopsy tract. The patient’s final imaging 4 months after initial diagnosis revealed the primary and secondary lesions had enlarged. Subsequently, the patient clinically deteriorated and died 7 months after initial diagnosis.

## Clinical presentation

A 44-year-old male presented to the emergency department with a 6-week history of headaches that had been initially worked up for a sinus infection. His headaches were worse with standing, bending, and lying down. He also reported having nausea, but denied vision problems, weakness, and numbness. In addition, the patient denied seizures and had not experienced any loss of consciousness. Past medical history was notable for hypertension and amyotrophic lateral sclerosis (ALS). His ALS had been diagnosed in 2010 and he had responded well to two stem cell injections in 2010 and 2011 as part of a clinical trial. Neurological examination was notable for new onset of motor weakness in the left upper and lower extremities.

## Investigations, Differential Diagnosis, and Treatment

**Figure 1.**
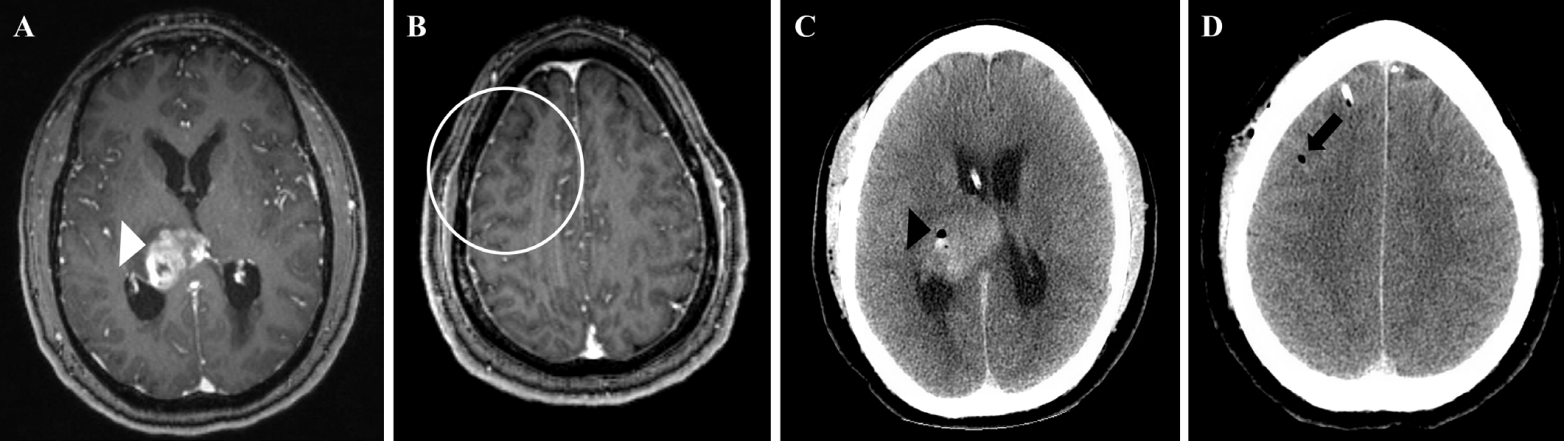
Imaging at the time of tumor diagnosis. Axial contrast-enhanced T1WMRI showing the primary lesion (white arrowhead), (A) and no secondary lesion in the right middle frontal gyrus (white circle), (B) at the time of diagnosis. Axial CT taken after patient underwent stereotactic biopsy of the primary lesion. Air in the tissue sampling location within the lesion (black arrowhead), (C). Higher level slice demonstrating air in the biopsy tract in the future site of the metastatic lesion (white arrow), (D).

MRI of the brain revealed a mass measuring 3.5 × 2.4×3.3 cm in the medial right thalamus-pineal region (Figure 1A). The lesion was hypercellular, hypervascular, and heterogeneously enhancing with a small amount of surrounding edema. The mass compressed the third ventricle, causing acute hydrocephalus. There was no secondary lesion observed on imaging in the right middle frontal gyrus at initial presentation (Figure 1B). The differential diagnosis based on the patient’s presentation, physical examination, and imaging were the following: high grade glioma, lymphoma, germ cell tumor, or a pineal parenchymal tumor.

Due the location of the lesion, the patient was not considered a candidate for surgical resection. Therefore, stereotactic biopsy was performed to establish a definitive diagnosis. The biopsy was performed via a right frontal trajectory to minimize functional morbidity and utilized an outer sheath. The inner needle was removed separately for each biopsy while the outer sheath remained in place. In addition, the patient had a ventriculoperitoneal (VP) shunt placed to address their acute hydrocephalus. Histopathology analysis revealed the mass to be glioblastoma (GBM). The molecular profile of the tumor was determined to be isocitrate dehydrogenase-negative and O-6-methylguanine-DNA methyltransferase indeterminate. Post-operative axial CT imaging (Figure 1C and D) demonstrated air in the biopsy tract at the site of the primary lesion and at a site near the entry point of the needle. No secondary lesion is observed on this post-operative CT. Subsequently, the patient began a 6-week course of radiotherapy along with concurrent temozolomide. The radiation treatment was prescribed to 60 Gy in 2 Gy per fraction to the T1 post-contrast enhancement and resection cavity, and 54 Gy in 1.8 Gy per fraction was prescribed to the T2 fluid attenuation inversion recovery (FLAIR) hyperintensity. During this time, the patient also enrolled in a clinical trial for patients with newly diagnosed GBM that involved receiving Belinostat, a histone deacetylase inhibitor, prior to initiation and during radiation treatment. Temozolomide was discontinued during the last week of the chemoradiation regimen due to thrombocytopenia. The patient completed radiotherapy about 2 months after initial presentation.

**Figure 2.**
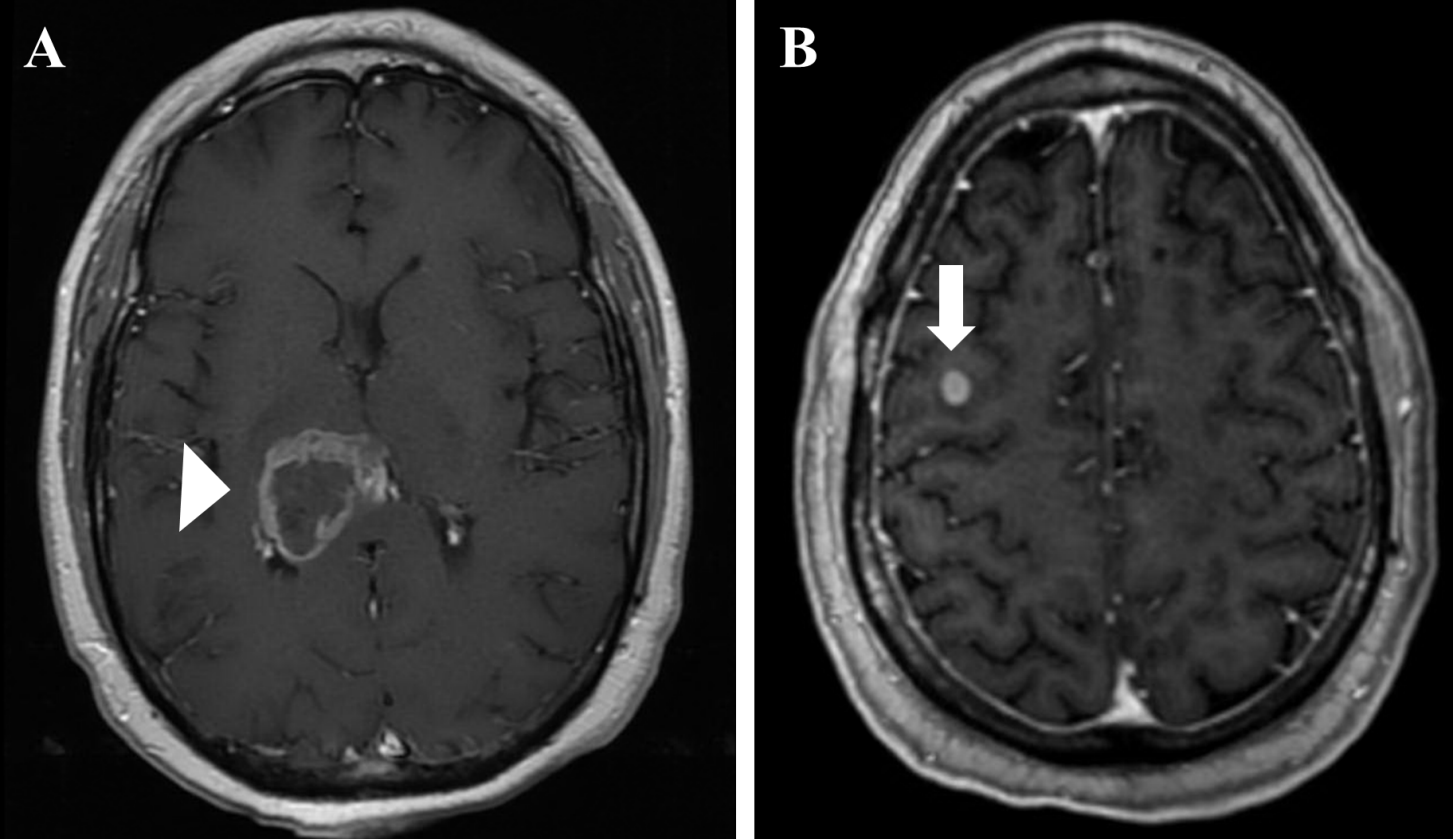
Follow-up imaging at 1 month after completing chemoradiation therapy (3 months since initial presentation).The primary tumor has modestly increased in size with central necrosis (white arrowhead), (A), and a secondary lesion along the biopsy tract in the right middle frontal gyrus is present (white arrow, (B).

**Figure 3.**
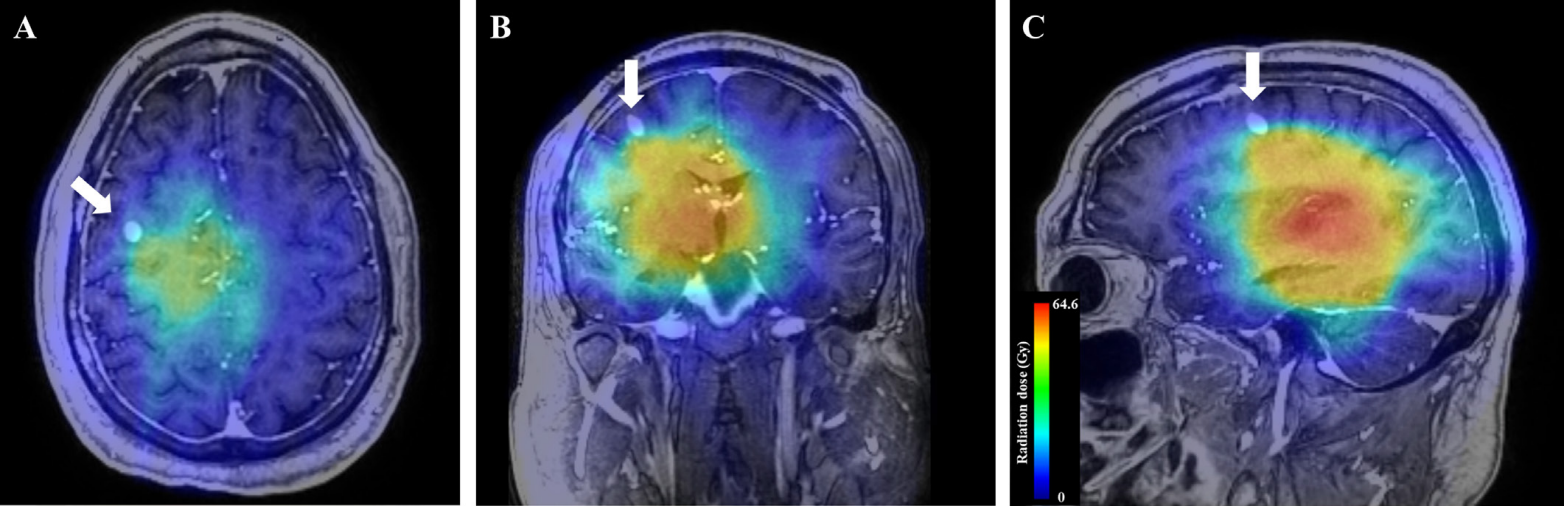
Radiation dose color maps superimposed on 3 month follow-up imaging. Dosing map superimposed on axial (A), coronal (B), and sagittal (C) T1W post-contrast MRI demonstrating the secondary lesion (white arrows). The distant nodule is outside the primary radiation field.

One month after the completion of chemoradiation (3 months since initial presentation), follow-up imaging revealed that the tumor in the right thalamic-pineal region had modestly increased in size (3.7 × 3.2×3.2 cm) with central necrosis (Figure 2A). However, a previously unseen hyperperfusing lesion (8 mm) was found in the right middle frontal gyrus along the biopsy needle tract (Figure 2B). Differential diagnosis for this mass were iatrogenic primary tumor metastasis secondary to the biopsy or infection. This lesion developed outside of the patient’s radiation therapy field (Figure 3) and was found to be in close proximity to the biopsy entry site, strongly suggesting it to be an intracranial metastasis of GBM. Given the high likelihood of a secondary site of GBM, the new nodule was treated with stereotactic radiosurgery without additional biopsy. The patient underwent standard stereotactic radiation treatment protocol consisting of immobilization with a thermoplastic brain mask, and on-boarding imaging was acquired daily prior to the treatment to confirm positioning.

## Follow-Up and outcome

**Figure 4.**
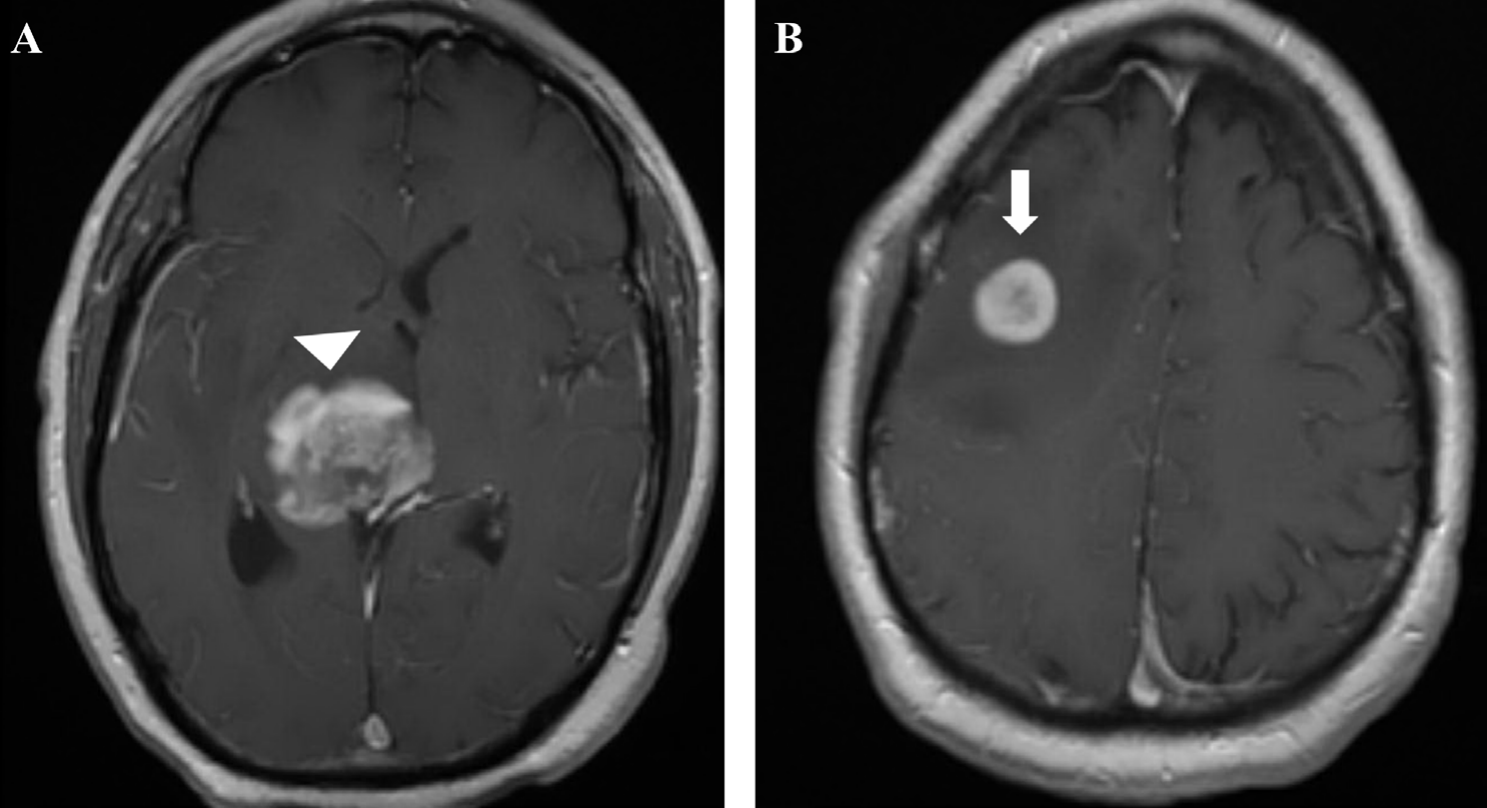
Follow-up imaging at 2 months after completing chemoradiation therapy (4 months since initial presentation). Axial contrast enhanced T1W MRI showing further growth of the primary tumor (white arrowhead), (A) and secondary tumor (white arrow), (B) along the biopsy tract.

Two months after completing chemoradiation therapy and 1 month after the secondary lesion received stereotactic radiosurgery (4 months since initial presentation), the patient reported having increased frequency of falls and significant gait instability. MRI at this time showed a considerable increase in size of both the primary and secondary lesions with a significant increase in edema (Figure 4), compared to the previous MRI done 1 month earlier (Figure 2). The patient’s dose of dexamethasone treatment was slowly increased from 4 to 24 mg daily. At a clinic follow-up appointment a few weeks later, his physical examination revealed further motor weakness in the left upper and lower extremities. This progression was considered progression and failure of temozolomide, and the patient was started on bevacizumab (Avastin) as a second line treatment for recurrent GBM;^1–3^ however, his platelet count continued to remain too low for adjuvant temozolomide. The patient remained clinically stable for the next 2 months, but deteriorated likely due to tumor progression. The patient died 7 months after initial diagnosis.

## Discussion

The ideal treatment protocol for GBM consists of surgical resection, followed by radiation therapy and concomitant temozolomide chemotherapy.^4^ However, when brain lesions are located in areas of the brain that cannot be surgically resected, such as the thalamus, stereotactic needle biopsy is an effective way for safely determining the diagnosis while minimizing complications.^5,6^ Complications for stereotactic biopsy are rare, with a reported rate of between 0.4 and 4%.^7,8^ The most common complications are hemorrhage, infection, and the risk of inconclusive results.^5,6^ Metastases resulting from seeding of the stereotactic biopsy needle tract is considered a rare complication. However, there have been case reports in the literature that discuss seeding of the biopsy tract in patients with brain metastases,^9^ pineoblastoma,^10,11^ anaplastic astrocytoma,^12^ and GBM.^13,14^ In addition, malignant seeding has been well documented in needle biopsy procedures for tumors in the liver,^15–17^ lung,^18^ breast,^19,20^ prostate,^21,22^ kidney,^23^ and GI tract.^24^

The secondary lesion that developed in this patient occurred along the biopsy tract and was outside of the radiation therapy field for the primary lesion (Figure 3). Any malignant cells spread along the needle course would not have received therapeutic doses of radiation, facilitating development of this secondary intracranial metastatic nodule. Pierallini et al and Buis et al reported similar cases of patients with brain tumors who received radiotherapy instead of surgery and went on to develop intracranial metastases associated with the biopsy needle tract outside of the radiation field.^13,25^ Pierallini et al reported that the volume of the primary tumor was unchanged between 3 and 6 months after radiotherapy treatment, but the metastatic lesion outside of the radiation field demonstrated further growth during the same time period.

There are several important conclusions from this case and similar reports of intracranial metastasis outside of the radiation field. First, radiologists interpreting tumor imaging and oncologists following brain tumor patients should be aware of the risk of developing secondary sites of disease along the biopsy tract. Second, generous clinical target volume expansions are typically added in treatment target delineation so that biopsy tracts are subsequently included, but there are rare cases such as this in which volume expansions still do not entirely cover the entire biopsy tract. Consequently, although spread along the tract is rare, extending the irradiated volume to include the entire biopsy tract in patients receiving radiotherapy after undergoing stereotactic biopsy of deep cerebral lesions could potentially be beneficial. This concept has been demonstrated in patients with malignant pleural mesothelioma, which has a high rate of malignant seeding along the biopsy needle tract.^26,27^ A clinical trial of 40 patients with malignant pleural mesothelioma that underwent needle biopsy via thoracoscopy showed that 100% (20/20) of the patients who received radiotherapy to the biopsy tract did not develop secondary lesions, while 40% (8/20) of the patients who did not receive radiotherapy developed metastases.^28^

This case adds to the existing body of literature that that malignant seeding of the needle tract during stereotactic biopsy for brain tumors is a possible complication, especially if the biopsy tract extends outside of the radiation field. Due to this tumor’s deep location in the thalamus, the biopsy tract was longer than what would be needed for superficial lesions. As a result, the radiation field focused on the primary lesion and left the superficial portion of the biopsy tract susceptible to malignant seeding. Therefore, clinicians should consider the location of the tumor and the length of the biopsy tract when planning radiation treatment. In clinical situations where lengthy biopsy tract is unavoidable, clinicians may consider expanding the irradiated volume to target the entire biopsy needle tract to decrease the risk of this complication.

## Learning points

1. Intracranial metastases associated with needle biopsy are a rare complication in brain tumor patients, but have the potential for impacting the patient’s clinical condition and outcome.
2. The region of the needle tract that is outside of the radiation field is at increased susceptibility to malignant seeding during stereotactic biopsy.
3. Prophylactic treatment of the biopsy tract with radiation therapy in addition to the tumor may decrease the risk of malignant seeding in patients with long biopsy tracts not likely to be covered by the primary field.
